# Synthetic calcium carbonate improves the effectiveness of treatments with nanolime to contrast decay in highly porous limestone

**DOI:** 10.1038/s41598-019-51836-z

**Published:** 2019-10-24

**Authors:** Radek Ševčík, Alberto Viani, Dita Machová, Gabriele Lanzafame, Lucia Mancini, Marie-Sousai Appavou

**Affiliations:** 1 Institute of Theoretical and Applied Mechanics of the Czech Academy of Sciences, Prosecká 809/76, Praha 9, 190 00 Czech Republic; 2 Elettra-Sincrotrone Trieste S.C.p.A., SS 14- km 163.5, Area Science Park, 34149 Basovizza (Trieste), Italy; 3 Forschungszentrum Jülich GmbH, Jülich Centre for Neutron Science JCNS at MLZ, Lichtenbergstraße 1, 85747 Garching, Germany

**Keywords:** Structure of solids and liquids, Characterization and analytical techniques

## Abstract

Three synthetized polymorphs of calcium carbonate have been tested in combination with the suspension of nanolime particles as potential consolidating agents for contrasting stone decay and overcome some of the limitations of nanolime agents when applied to substrates with large porosity. The modifications induced in the pore network of the Maastricht limestone were analyzed with microscopy and in a non-invasive fashion with small angle neutron scattering and synchrotron radiation micro-computed tomography. A reduction in porosity and pore accessibility at the micrometric scale was detected with the latter technique, and ascribed to the improved pore-filling capacity of the consolidation agent containing CaCO_3_ particles. These were found to be effectively bound to the carbonated nanolime, strengthening the pore-matrix microstructure. Penetration depth and positive effect on porosity were found to depend on the particle size and shape. Absence of significant changes in the fractal nature of the pore surface at the nanoscale, was interpreted as indication of the negligible contribution of nanolime-based materials in the consolidation of stones with large porosity. However, the results indicate that in such cases, their effectiveness may be enhanced when used in combination with CaCO_3_ particles, owing to the synergic effect of chemical/structural compatibility and particle size distribution.

## Introduction

A large part of tangible cultural heritage is made of stone. In consequence of its exposure to weather, every stone is subjected to deterioration. Stone decay is a major cause of loss of cultural heritage and has a high social and economic impact. Many efforts have and are still devoted to devise solutions to limit or prevent the damage. With the development of methods and procedures for conservation, it has become evident that, to be effective, every treatment of the stone must be tailored to each specific case^1–3^. Actually, when active conservation through consolidation is concerned, the requirements to be fulfilled are not limited only to strength improvement, but must include chemical and physical compatibility of the consolidation agent with the treated stone (e.g. similar thermal expansion, elastic modulus, assure similar permeability), long-term stability and performance (avoiding self-deterioration, colour changes, by-product formation)^4,5^. It is therefore not surprising that most of the consolidation agents available on the market show drawbacks and the design of new products is a very active field of research^6–9^.

In case of stones composed by calcium carbonate (CaCO_3_), such as limestone rocks, lime based agents fulfill the requirements of compatibility, thanks to the conversion of Ca(OH)_2_ into calcium carbonate (the so-called carbonation reaction)^10,11^. Nonetheless, lime water, which is frequently employed at the scope, has several disadvantages, such as the low concentration of Ca(OH)_2_, which imposes the exposure of the treated object to high volumes of water^12^. Recently, colloidal suspensions of nanolime were introduced as a suitable alternative to lime water^8,13^. These nanosized Ca(OH)_2_ particles suspended in alcoholic solvents have been proposed for the treatment of different cultural heritage objects^13,14^ and considered promising as consolidation agents^10,15,16^. Nanolime suspensions allow for a better penetration, faster carbonation and higher loads of Ca(OH)_2_ into the treated volume^17^. Compared to aqueous media, alcohol-based systems reduce the rate of agglomeration^18^. Alcohol may leave the consolidated material by evaporation, however, it was shown that in some cases, Ca(OH)_2_ partially transformed into calcium alkoxides (calcium ethoxide or calcium isopropoxide)^3^, impairing the effectiveness of the treatment. Despite many advantages, still some drawbacks persist^4,12,19,20^. In general, the penetration strongly depends on the physical-chemical characteristics of the stone (e.g. nature and extent of deterioration, microstructure, lithology). Large pores are not filled, and their surface is only partly covered with a thin layer of calcium carbonate. A well-known problem, especially evident in highly porous stones, is the accumulation of the Ca(OH)_2_ nanoparticles (30 to 100 nm^21^) at, or just beneath, the surface of the treated material as a consequence of the high volatility of the used solvent, which favors the partial back-migration of the nanolime^22,23^. To date, none of the proposed solutions to contrast this effect^4,19,20,24,25^ appear as conclusive.

In this work, we report on the synergic effect of calcium carbonate polymorphs, synthesized in the laboratory (calcite, aragonite and vaterite), in combination with nanolime for the consolidation of a highly porous stone (>50%), the well-known Maastricht limestone (ML)^19,26^.

The effectiveness of the treatment has been assessed with scanning electron microscopy (SEM) and quantitative analysis of the stone pore network. In order to overcome the limitations of the fluid inclusion techniques, not last the damage of sample microstructure due to the applied pressure, in the latter case, measurements have been conducted by combining two fully non-invasive techniques, namely, small angle neutron scattering (SANS) and synchrotron radiation micro-computed tomography (SR-µCT).

## Materials and Methods

### Raw materials and sample preparation

The ML is an Upper Cretaceous soft and highly porous bioclastic calcarenite, belonging to the Maastricht formation, with extensive outcrops in the southern Limburg province between Belgium and The Netherlands. This material has been widely used from the Middle Age to the Renaissance and is still quarried nowadays (Sibbe Quarry, The Netherlands), mainly for restoration purposes^27^. It is mainly composed of skeletal elements of ostracodes, foraminifera, sponges, bryozoa and brachiopods, all cemented with calcite spar, with minor amounts of glauconite, quartz, iron oxide and other opaque minerals^28^. The susceptibility to weathering, is largely due to its high porosity, and it is only in part attenuated by the development of a thin hard surface layer formed by re-crystallization of calcium carbonate^26,29^.The majority of the pores have size comprised between 125 and 250 μm, with an average porosity around 50% (the values depend on measurement techniques)^28,30^. Reported values of average pore size from mercury intrusion porosimetry (MIP) and X-ray micro-computed tomography (μCT), were 39.0 μm and 28.4 μm, respectively^31^.

**Figure 1:**
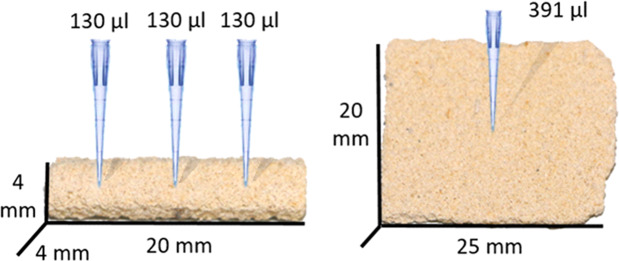
Visualization of application of consolidation agents for SR-μCT (left) and SANS measurements (right).

Two different shapes of testing specimens have been prepared: prisms of about 4 × 4 × 20 mm for SR-µCT measurements and slabs about 20 × 20 mm with thickness of about 2 mm for SANS measurements (see Fig. 1).

### Application of consolidation agents

All samples were consolidated in two-steps: first, a suspension of nanolime Calosil E25 (IBZ-Salzchemie, Germany, concentration = 25 g.L^−1^) with CaCO_3_, followed by the same amount of suspension of pure nanolime. The applications of consolidation mixtures is graphically represented in Fig. 1. Due to different size of testing specimens, the amount of nanolime suspension was 3 × 130 µL (corresponding to 3 × 3.250 mg of Ca(OH)_2_) and 391 µL (corresponding to 9.775 mg of Ca(OH)_2_), for the ML samples employed for the SR-μCT and SANS experiments, respectively (see Fig. 1).

Synthetic calcite, vaterite and aragonite, were synthesized as previously described^32,33^. Their characterization was reported in^34^. In order to decrease grain sizes, vaterite and aragonite were milled for 3 and 5 minutes, respectively, using Mini-Mill Pulverisette 23 (Fritsch) operated at 30 oscillations per minute. Three different concentrations of each of the CaCO_3_ polymorphs were employed for the SR-µCT measurements: 5 wt%, 15 wt% and 50 wt%, corresponding to 0.493 mg, 1.470 mg and 4.903 mg, respectively. Concentrations 5 wt%, 15 wt% were employed in the SANS measurements. The samples have been measured after 6 weeks of storage in climatic chamber under controlled conditions (t = 20 ± 1 °C, RH = 65 ± 5%). After this period, according to previous results^35^, complete transformation of nanolime into CaCO_3_ should be achieved. Samples treated with nanolime E25 only, and untreated samples of limestone, were used as reference materials. Two sample replicates for each of the treatments were produced and results averaged.

### Analytical methods

The naming convention adopted in this paper for the samples is XXX_Y_Z, where X identifies the type of treatment (REF for untreated ML (reference), E25 for nanolime, ARA for nanolime + aragonite, CAL for nanolime + calcite, VAT for nanolime + vaterite), Y the concentration of the CaCO_3_ polymorph (5, 15, 50), and Z the sample replicate (1 or 2).

#### Scanning electron microscope (SEM) analysis

Sample texture and microstructure have been studied with scanning electron microscope (SEM), employing a FEI QUANTA FEG 450 instrument equipped with backscatter electron, secondary electron and energy dispersive (EDS) detectors. The samples were observed at 20 kV accelerating voltage as broken fragments and in polished cross-section, after coating with 5 nm thick gold film.

#### Particle size analysis

Grain size distribution of CaCO_3_ polymorphs was determined by laser granulometry (Cilas 1090 LD). Prior to measurement, the powders were dispersed in isopropanol alcohol, circulating for 15 s, and sonicated for 60 s. The standard procedure during measurement was set to 120 rpm circulation with 200 rpm stirring speed and 30 s measurement time.

#### Phase-contrast SR-µCT measurements and image analysis

High resolution SR-µCT measurements were performed at the SYRMEP beamline of the Elettra Sincrotrone Trieste facility in Basovizza (Trieste, Italy). The samples were mounted on a rotating stage and imaged in local or region-of-interest mode^36^ employing a filtered white X-ray beam (1.5 mm Si + 1 mm Al). For each experiment, 3000 radiographic projections over a total angular range of 360° were acquired with an exposure time/projection of 1 s. Two partly overlapping volumes for each sample were covered by displacing the sample vertically.

The detector used was an air-cooled 16 bit sCMOS camera (Hamamatsu C11440-22C) with a 2048 × 2048 pixel chip. The effective pixel size was set at 1.4 μm × 1.4 μm. In order to enhance the visibility of phases with similar linear attenuation coefficients and the pore edges, we worked in propagation-based phase-contrast mode, setting the sample-to-detector distance to 150 mm^37,38^.

The tomographic reconstruction of the SR-µCT images was performed by the SYRMEP Tomo Project software developed at Elettra^39,40^, using the Filtered Back Projection algorithm^41^. A single distance phase-retrieval algorithm^42^ was applied to the projection images in order to improve the reliability of quantitative morphological analysis and enhance the contrast between the pores and the solid. To this aim the optimal ratio *γ* = *δ*/*β* between the real (*δ*) and imaginary (*β*) parts of the refractive index was fixed to 20.

Processing and quantitative analysis of the reconstructed volumes were performed by means of Pore3D software library^43^ (http://www.elettra.eu/pore3D) developed at Elettra. The freeware software Fiji^44^ was adopted to visualize the reconstructed or processed bi-dimensional (2D) slices.

Image segmentation was adopted in order to isolate the pores from the matrix of the samples. An anisotropic diffusion filter facilitated the pore edge recognition^45^. Segmentation was then performed using the automatic Otsu’s method^46^. Filtering and segmentation were performed on the whole investigated volume, whereas the more computationally intensive steps of the quantitative image analysis have been performed on a suitable volume of interest (VOI). The significance/representativeness of the VOIs has been assessed by determining the Representative Elementary Volumes (REVs) for each sample. This approach, which has become standard in the analysis of SR-µCT data of porous media^47^, is based on the definition of the REV as the volume in which the measurements of a sample property (i.e. porosity) is scale-independent and accurately represents a larger volume, that is, the whole investigated sample^48^. The REV was identified with plots of porosity and connectivity parameters, calculated on progressively larger concentric cubic volumes^47,49,50^ (an example is provided as Fig. S1). Larger VOIs (1800 × 1800 × 1948 voxels), corresponding to a volume of 17.3 mm^3^, were chosen as representative. Therefore, the VOI did not include the sample surface.

The following parameters were obtained thanks to the modules included in the Pore3D software library: porosity (Φ) [%]); specific surface area of pores (*S*_*V*_ [mm^−1^]); fractal dimension (*D*_*F*_) of the surface, according to the fractal theory^51^ employing the box-counting method; frequency distribution of pore volumes. Further analysis of pore accessibility ratio was conducted applying the Pore Size Distribution (PSD) plugin^52^, implemented in Fiji^44^. The same procedure, the ratio between the constrained PSD obtained from the MIP simulations and the unconstrained PSD obtained from 3D continuous approach, as further described in^53^ was applied for calculation of pore accessibility ratio. The MIP simulations were calculated using intrusion only from the side of application of consolidation agents.

#### Small angle neutron scattering (SANS)

SANS data were collected from samples with thickness ranging from 2.1 to 2.6 mm on a 2-D detector as intensity (*I*) in function of the scattering angle (defining the angular deviation of the scattered beam with respect to the incident direction), at the KWS-2 instrument^54,55^ operated by the Jülich Centre for Neutron Science (JCNS) at the Heinz Maier-Leibnitz Zentrum MLZ (Garching, Germany). The *Q* range 0.0015–0.45 Å^−1^ was covered by merging data collected at wavelength *λ* = 5.0 Å with sample-to-detector distance 1.105 m and 7.605 m, and at wavelength *λ* = 10.0 Å with sample-to-detector distance 19.505 m. The 2-D raw data have been corrected for beam attenuation (according to the measured sample thickness), the scattering from the empty cell, the electronic and background noise. Intensity was calibrated against a Plexiglas standard material to set the data to absolute scale. No correction for multiple scattering effects has been applied, since sample thickness was chosen in order to minimize it (calculated transmission >90%)^56^. Instrument data analysis and background subtraction was carried out using the QtiKWS software provided by JCNS.

SANS data treatment and analysis: The SANS technique has been largely employed for the microstructural characterization of dense porous systems, such as ceramics and rocks^57–60^. In a SANS experiment, the fluctuation of neutron scattering length density (a measure of the interaction of neutrons with matter) within the sample, is measured. The signal is generated by the scattering contrast between the components in the investigated volume, which is defined by the beam spot size (around 1 cm), and the sample thickness. The SANS technique is completely noninvasive and allows for gaining access to both open and closed porosity in the investigated size range. In porous solids, the microstructure is usually described through the two- phase approximation (i.e. matrix-pores); the measured scattered intensity is proportional to the scattering contrast, with the pores showing a much lower neutron scattering length density than the solid^56^. Since the Maastricht limestone is essentially composed by calcium carbonate and after the treatments only new calcium carbonate should be added, this assumption can be considered valid. The scattering contrast has been calculated using the mineralogical composition^61^ obtained from quantitative phase analysis using the Rietveld refinements of X-ray powder diffraction data. Results indicated 99.7 wt% calcite and 0.3 wt% quartz. Mg content in calcite has been refined to 0.6 mol% of MgCO_3_. This value is in good agreement with the one obtained by plotting cell parameters on calibration curves from biogenic calcite^62^.

Following a standard approach^63^, the effect on the scattering contrast of the added amount of consolidation material resulted negligible and was not considered.

Details of the SANS theory can be found elsewhere^64^. Here few basic concepts are summarized.

The scattering curves (1-D) of *I* vs. momentum transfer (*Q*) are obtained by radial averaging of the data in the 2-D patterns collected at the detector. *Q* is defined as:

1 $$Q=\frac{4\pi }{\lambda }\,\sin \,2\theta $$

where 2θ is the scattering angle.

In our case, the 1-D curves have been derived by radial integration over 360°. The curve contains information about the shape and size distribution of the scatterers (in our case, pores). Although analytical expressions exist to account for different pore shapes, the complex microstructure of porous solids is usually described assuming spherical shape for the pores^60^. This form factor has been adopted to retrieve the volume weighted pore size distribution through the fit of the SANS curves with the software McSAS^65^, which implements a fitting procedure based on the Monte Carlo method.

In the SANS experiment, the size of the pores is inversely proportional to *Q*, and at high *Q* values $(Q\gg 1/R)$, where *R* is the pore radius, the shape of the scattering curve depends solely on the characteristics of the pore surface. Therefore, a change in the slope observed in the SANS curve, when plotted as log *I*(Q) vs. log *Q*, indicates a change in the nature of the scattering surface, corresponding to different length scales. The analysis of the slope of the scattering curves is a common approach to the study of porous solids, since it enables the description of the quality of pore surface and identification of scattering regimes^66^.

In general, within one scattering regime, the curve obeys a power law of the type:

2 $${\mathrm{lim}}_{Q\to \infty }I(Q)=B{(\frac{1}{Q})}^{n}$$

In Eq. 2, *B* is a coefficient proportional to the scattering contrast *K*, to the volume of sample irradiated, and to the number density of voids. The exponent *n* allows for the fractal description of the structure of the scattering objects. For mass or volume fractals, the fractal dimension *D* = *n*, and 1 < *n* < 3. For surface fractals, the fractal dimension *D*_*S*_ = 6 − *n* and 3 < *n* < 4. In case of porous solids exhibiting continuous change in scattering density and with smooth boundaries, the exponent *n* can also assume values higher than 4. A special case is represented by solids with sharp and smooth surfaces, for which *n* = 4 and the Porod law is obeyed^67^. Equation 2 thus, becomes:

3 $$I(Q)=2\pi {S}_{V}{K}^{2}{(\frac{1}{Q})}^{4}+C$$

where *K* is the previously mentioned scattering contrast within the sample, that can be calculated from the chemical composition, and *S*_*V*_ is the scattering surface area per unit volume of sample irradiated by the beam. *C* is the constant incoherent background. In dense systems, within one scattering regime, in the limit of high *Q*, the scattering curve obeys the Porod law and the scattered intensity becomes proportional to *S*_*V*_, irrespective of the shape of voids. However, to trust this assumption, the slope must extend for at least one full magnitude in *Q*.

## Results and Discussion

**Figure 2:**
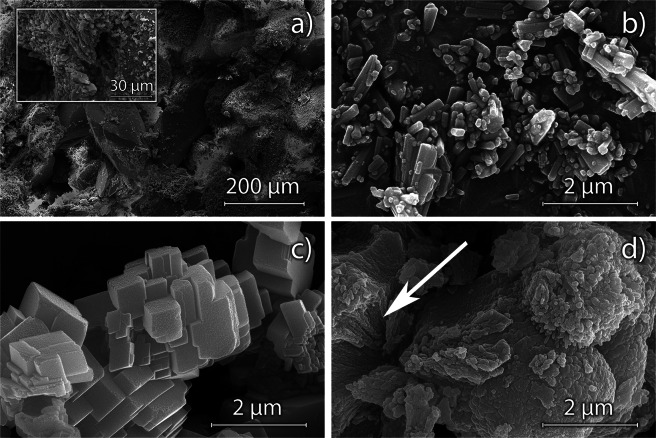
SEM micrographs of the Maastricht limestone (**a**), exhibiting large calcium carbonate crystals (center of field of view) cemented by small individuals, as illustrated in the inset. CaCO_3_ polymorphs employed in the experiment: aragonite (**a**); calcite (**b**); vaterite (**d**).

Figure 2a shows the microstructure of the Maastricht limestone as seen under the SEM. Calcium carbonate clasts span a wide range of sizes, from nanometric to ∼500 μm. Most of the visible pores are from 125 μm to about 250 μm in size. Figure 2b–d illustrate the grains of the synthesized CaCO_3_ polymorphs employed in the experiment. It can be noticed that milling effectively decreased the size of aragonite (Fig. 2b), reducing the natural crystal elongation (its crystal habit is, in fact, needle-like), facilitating its penetration into the pore structure of the limestone. Calcite (Fig. 2c) is characterized by grains formed by the coalescence of rhombohedral individuals. Vaterite (Fig. 2d) was present as sub-spherical particles few μm in size, formed by the coalescence of smaller nanoparticles^32^. The internal structure of the vaterite grains can be appreciated in their fractured sections (arrow in Fig. 2d).

**Figure 3:**
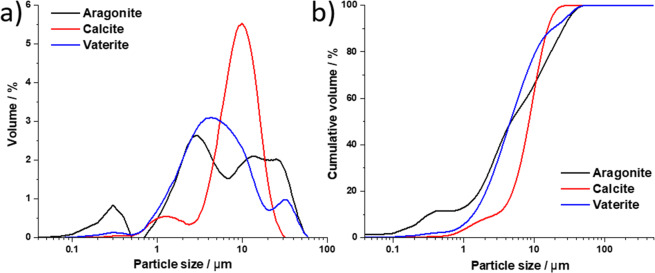
Density distributions (**a**) and particle size cumulative volumes (**b**) of synthetic anhydrous CaCO_3_ polymorphs used in consolidation agents’ mixtures.

The grain size distributions of CaCO_3_ polymorphs employed in the consolidation mixtures, are illustrated in Fig. 3. The average sizes, expressed as d_50_ (average diameter at 50% of values), were 8, 4 and 4 µm, for synthetic aragonite, calcite and vaterite, respectively. The shapes of the distributions differ, with aragonite exhibiting a tri-modal distribution with local maxima at 0.3, 2.7 and 10.5 µm, and calcite and vaterite exhibiting bimodal distributions with local maxima at 1.3, 8.3 and 4.0, 12.3 µm, respectively.

**Figure 4:**
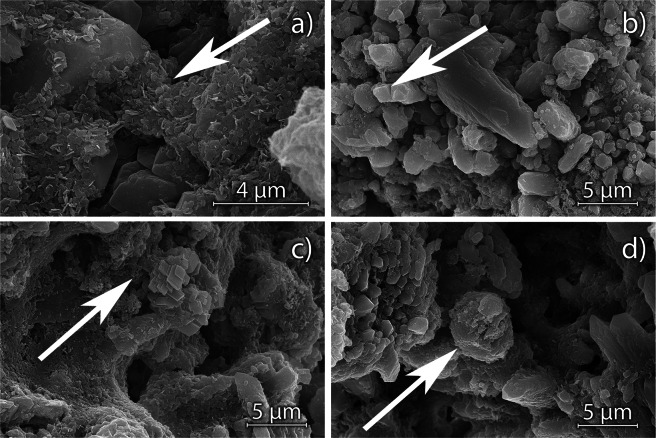
SEM micrographs illustrating the effect of treatments with the pure nanolime in the sample volume (**a**), ARA15 (**b**), CAL15 (**c**) and VAT15 (**d**).

Figure 4 illustrates the effects of the treatments with consolidation agents on the limestone microstructure, as observed under SEM of internal fragments. In case of pure nanolime (Fig. 4a), examples of masses formed by the accumulation of nanoparticles, converted into calcium carbonate and bridging between grains in the matrix (indicated by arrow), have been detected. Notably, the large pores are not filled with the consolidant. When aragonite is added to the nanolime, episodes in which large particles of aragonite obstruct pores have been observed, as illustrated in Fig. 4b. Crystallization of nanolime on calcite particles was visible (Fig. 4c arrow), indicating that the presence of CaCO_3_ introduced with the nanolime is of help in filling the pores too big to be filled by nanolime alone.

Similar condition has been observed for vaterite (Fig. 4d). Vaterite particles surrounded by carbonated nanolime, with a variable degree of connection with the original sample matrix, can be observed in the sample (arrow).

SEM observations are indicating that polymorph addition is beneficial, likely because of the synergic effect of chemical/structural compatibility and particle size distribution, which may promote CaCO_3_ crystallization and improve the pore-filling capacity, respectively. The latter aspect is confirmed also in the cross-sections of the samples, reported in Figs S2–S4, where the synthetic CaCO_3_ particles were detected well inside the sample volume. As expected, the depth of penetration was found to depend upon their morphology (dictated by crystal habit and grinding process) and size distribution, in relationship with the characteristics of the pore network of the treated stone. The penetration of larger particles of aragonite was limited to about 500 µm, with smaller particles penetrating deeper into the matrix. For example, Fig. S2 illustrates an aragonite particle (of about 4.3 µm length, 2.7 µm thick) 190 µm from the surface. Slightly smaller particles, around 4.0 × 1.5 µm, were detected at 450 µm from surface. Some particles were observed also at higher depths, e.g. 2.3 mm, such as the one shown in Fig. S2c. However, the needle-like shape of the aragonite particles hampered their penetration in the pore network. The mixtures containing calcite particles, more isotropic in shape, were observed more in depth into the matrix and, as shown in Fig. S3, some of them penetrated up to 2 mm in the sample. Notably, calcite particles effectively filled some large pores, they were attached to the Maastricht limestone through the carbonated nanolime particles, which partly covered them (Fig. S3b–d). In a recent work^68^, consolidation with aqueous dispersion of calcite nanoparticles with addition of polymeric dispersant evidenced a limited penetration depth, with less than 10% of particles detected at 0.2−0.5 mm. It might be argued that, unlike larger grains, the calcite nanoparticles suffered from back-migration effect.

In case of vaterite, the particles could be detected up to 2.2 mm (Fig. S4). Their identification is complicated by the morphological alterations induced by milling, however, some examples are reported in Fig. S4b–d. Also in this case, the vaterite particles exhibited good compatibility with both the nanolime and the sample matrix, with examples of bridging between them (Fig. S4c).

**Figure 5:**
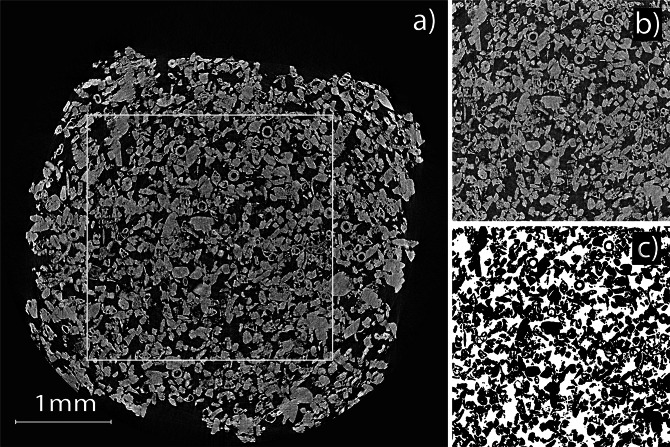
Axial SR-μCT view of the sample CAL_5_1 (**a**). VOI is indicated as white contour. The same axial view of the VOI before (**b**) and after (**c**) filtering and segmentation.

Figure 5 depicts an example of the axial view after reconstruction and the resulting segmented slice of the VOI in the sample CAL_5_1, obtained from SR-μCT. Several regular features, testify the abundance of microfossils. As expected, only one source of contrast can be observed, that is, between CaCO_3_ and voids in the matrix.

**Figure 6:**
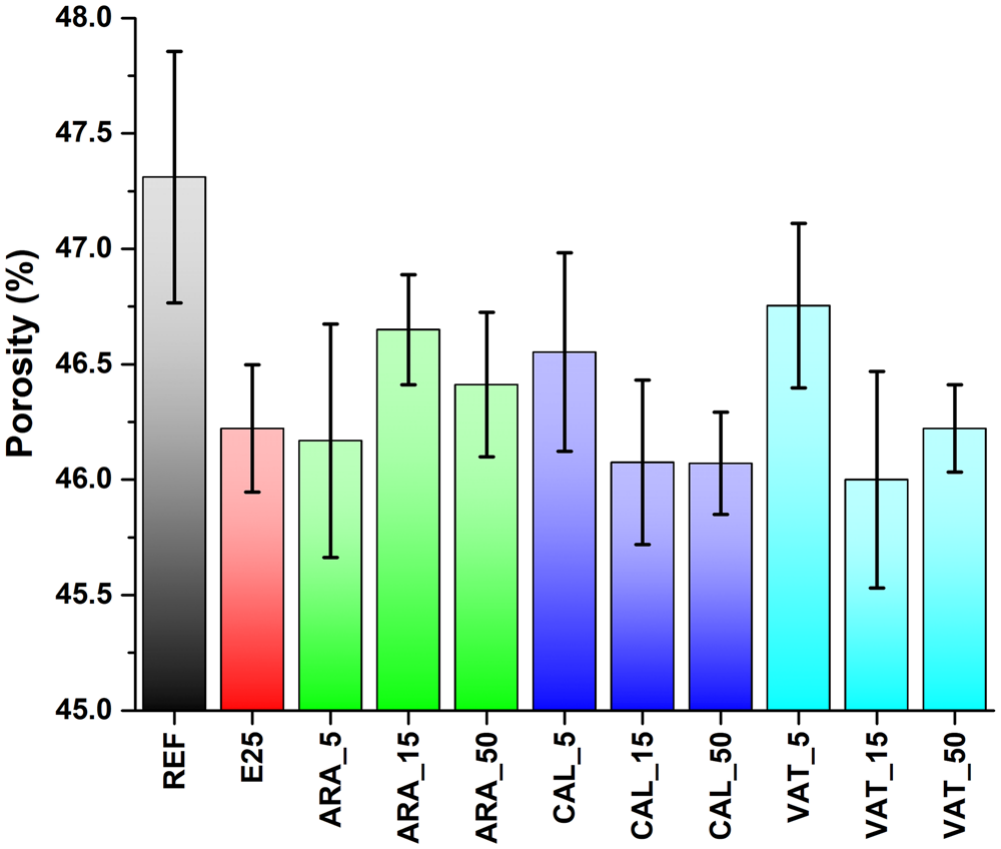
Porosity retrieved from 3-D image analysis of SR-μCT data on treated samples, as indicated. Standard deviation of values from different VOIs are reported.

Values of porosity obtained from 3-D quantitative image analysis are illustrated in Fig. 6. The investigated volume does not include the sample surface (as exemplified in Fig. 5), therefore, the results are not biased by the accumulation of CaCO_3_ particles at the sample surface, an effect observed under SEM and extensively documented in the literature^2,8,12,13^. The untreated sample, shows the highest value, as expected. This value is in line with literature data^19,31,32^ indicating that large part of the porosity falls within the range of sizes accessed in the SR-μCT (>2.75 μm^3^). When the standard deviation (std) is considered, the effect of the consolidation treatment appears small. However, it must be argued that the std, resulting from the average of different VOIs and 2 replicates, largely reflects the natural variability of the stone. This is confirmed by the similar values of std observed for untreated and treated specimens.

The quantitative analysis of the tomographic data evidenced that, apart from the dependence from the size distribution of particles discussed above, the powder load plays also a role. The addition of 5 wt% of calcite and vaterite to the suspension seems less effective than the application of nanolime alone (sample E25), whereas, the porosity decreased after treatment with 15% of powder. However, the effect of increasing the load from 15% to 50% is in both cases negligible, suggesting that a saturation level is reached and no more particles are entering the pore system. Aragonite exhibits a different behaviour, since the treatment with 5% of powder is more effective than with higher loads. This could be, as already discussed above, due to the peculiar elongated shape of the particles, which might have hindered their penetration, and likely also to the nanolime, favouring particle accumulation and obstruction of the pores at the very surface of the sample. Such effect, which is of detriment to the properties in this case, could be beneficial in others, for example to increase the surface compactness or as protective coatings of cultural heritage objects^69^.

Overall, the consolidated samples exhibited a decrease in detected porosity from 2 to 3% with respect to the untreated one (REF).

**Figure 7:**
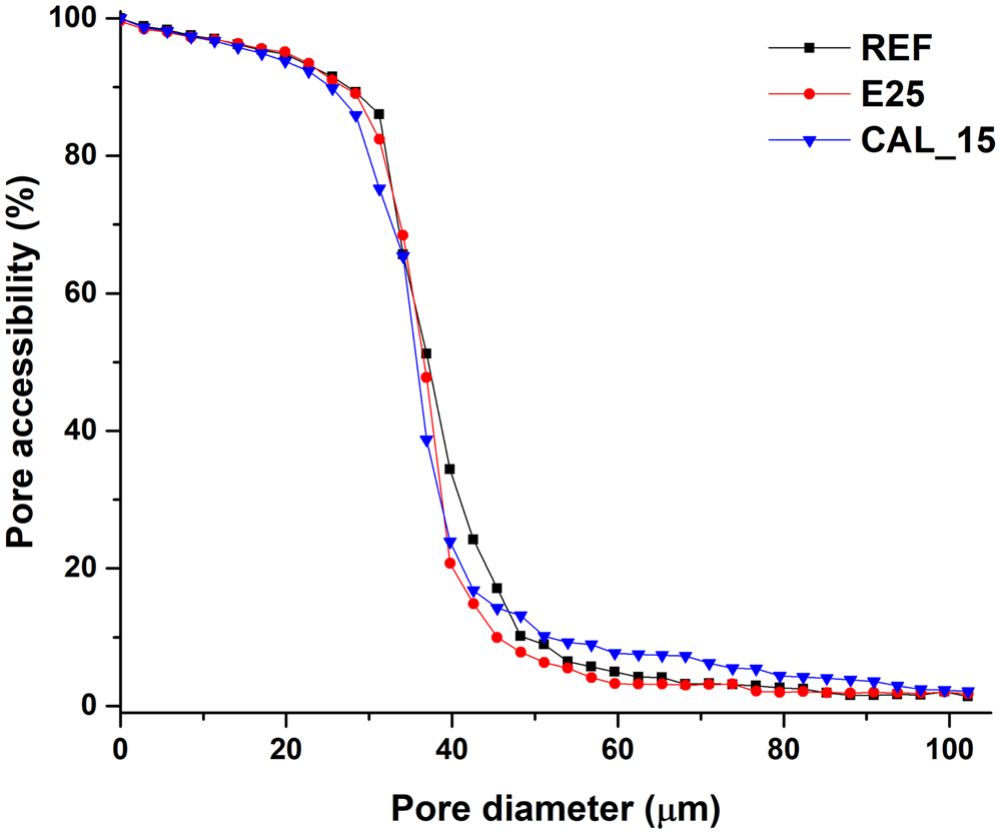
Pore accessibility ratio of the intra-aggregate pore space for the spherical entities. For sake of clarity only samples REF, E25 and CAL_15, are displayed. The others samples are reported in Fig. S5.

As a further confirmation of what discussed above, the pore accessibility ratio investigation was undertaken for the samples with nanolime additivated with 15% of synthetic CaCO_3_. Figure 7 shows that the access to the pore network from outside of the VOI for spherical objects with diameter > 25 μm, is hindered for calcite, compared to the samples REF and E25. The same holds for aragonite and vaterite (corresponding plots are reported in Fig. S5, together with the 3D continuous approach and 3D MIP simulation plots used in the calculation, Fig. S6, according to^53^), in agreement with the results of porosity illustrated in Fig. 6.

The detected reduction in porosity might be considered small, but it is in line with other results from the literature^70–72^. In fact, the results should be considered good, when taking into account the previously discussed exclusion of the surface layers (for a thickness ranging from 0.2 to 1 mm, depending on the sample), which exhibits the highest concentration of consolidation agent. In any case, compare results of porosity obtained from different techniques is difficult, because SR-μCT and SANS are sensitive to both open and closed porosity. The latter is not accessible to consolidation treatments and is relatively high in the Maastricht limestone, due to the presence of many fossils. In this respect, the pore accessibility ratio better reflects the impact of the treatments, since the closed porosity is not considered in the calculation. The analysis may also help in explaining the changes in some dynamic properties of relevance for the deterioration processes (e.g. hygroscopic behavior, water absorption). In any case, in literature it is well documented that even a small reduction in porosity may have a high impact on mechanical properties when applying nanolime^70,71^ or lime water^72^. For example, the splitting tensile strength of consolidated friable lime plaster was found about 45% higher^71^, with a decrease in porosity of 0.2%. An increase in compressive strength of about 30% and drilling resistance of about 76% was observed for a sample exhibiting a decrease in porosity of about 3% after treatment^70^. In this view, the consolidation formulations which reduced the porosity most effectively, ARA_5, VAT_15, CAL_15 and CAL_50, seem to be good candidates for improving the properties of the investigated stone material.

In agreement with what discussed above, the graph of the cumulative pore size distribution, obtained from the quantitative analysis of the SR-μCT data, is not very informative for most of the samples. Fig. S5 reports an example comparing samples CAL_50, CAL_5 and REF.

The values of *S*_*V*_ were comprised between 41.1 and 41.8 mm^−1^ and did not show any recognisable trend. The impact of the treatments on the roughness of the internal sample surface, as described by fractal dimension, *D*_*F*_, was not detectable, since all values were centred at 2.72 ± 0.01, indicating a surface fractal.

**Figure 8:**
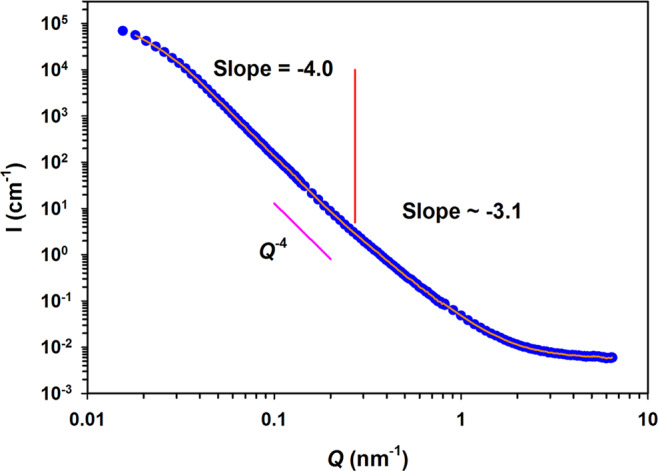
Example of normalized SANS curve for the sample CAL_5_1. Best fit to the curve is shown as continuous line. Error bars within symbols. *Q*^−4^ slope is reported.

When considering the SANS results, Fig. 8 depicts an example of log *I*(*Q*) vs. log *Q* plot obtained by radial averaging of the data in the 2-D patterns collected at the detector (the SANS curves of all samples are compared in Fig. S8). The homogeneous distribution of the intensity at the detector (see Fig. S9), indicates the absence of microstructural anisotropy or oriented texture, sometimes observed in rocks as a consequence of diagenetic processes.

The result of the best fit obtained using the Monte Carlo method is also illustrated in Fig. 8. The SANS normalized intensity is dominated by the pore/matrix interface, and, if the density of the solid matrix is the same for all the samples, a condition fulfilled in the present case, the normalized integrated intensity under the SANS curve is proportional to the amount of the scattering surface. The SANS curves overlap (see Fig. S8), indicating that the differences in fine porosity (covered in the experiment) are very small.

The analysis of the scattering curves mentioned in section 2.3.4.1, was performed adopting single- and two-slope fit models. Results (see Fig. S10) indicated a change in slope for all the curves at around 0.26 nm^−1^, separating two scattering domains with different length scales, therefore, pointing to a different nature of the surface. At low *Q*, for length scales 210−24 nm (calculated as 2*π*/*Q*), the slope is −4.0, typical of a Porod regime with smooth pore surface. At high *Q*, for length scales <24 nm, the slope is −3.1, which can be interpreted as a surface fractal with very rough surface^58^. It is worth noting that the entity of this change in slope is modest, moreover, some degree of uncertainty in the determination of the slope might arise because of the merging procedure during data reduction.

**Figure 9:**
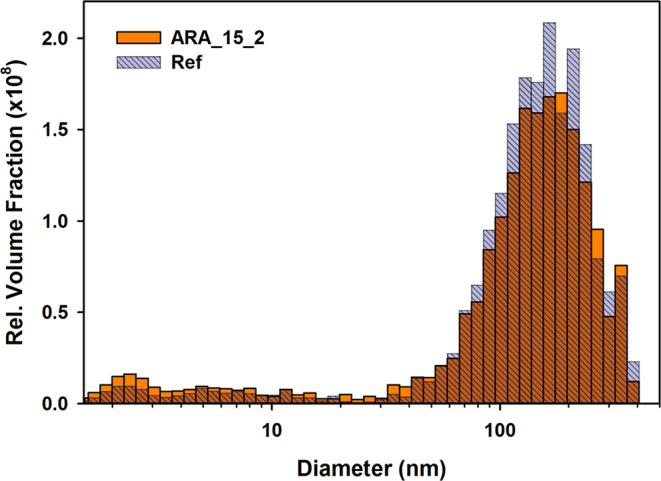
Volume-weighted size distribution of pores (*d* = pore sphere diameter) associated with the Monte Carlo fit of SANS curves for the sample ARA_15_1.

At low *Q* the curves flatten, this knee is indicating a tendency to a Guinier regime^64^, related to the distribution of sizes and shapes of scattering objects. As observed in other multiscale systems, such as sintered ceramics, cements and rocks^58,60,73^, it likely connects two domains. One, outside the investigated range, which could be the one detected with SR-μCT (exhibiting a *D*_*F*_ = 2.72), and the Porod regime with slope 4. The characteristic length scale of this Guinier regime can be obtained through a fit with a generalized Guinier-Porod model^74^, which is a linear combination of the two. The shape factor of the model was close to 1, compatible with an elongated, cylindrical shape. Therefore, this value was fixed. The derived radius of gyration *R*_*g*_, is related to the radius of the pores giving rise to the Guinier regime, assuming random orientation, by $R=\sqrt{2}{R}_{g}$^75^. No significant differences were observed in *R*_*g*_, whose absolute values ranged from 47 to 50 nm, indicating that this is a feature of the porous network not affected by the treatment. Figure 9 illustrates one example of pore size distribution associated with the fit of the SANS curves. It is visible that consolidation mainly reduced the pore size distribution in the range from 80 to 300 nm. The changes in the region from 2 to 20 nm were found to be negligible in all cases.

Numerical results obtained for the samples are reported in Table 1. It can be noticed that, at the scale accessible to SANS, the porosity doesn’t show significant differences between the samples, suggesting the negligible impact of the treatments with the synthetic CaCO_3_. It is worth noting that the absolute value of SANS porosity is a small fraction of the total porosity (less than 5%) and includes the closed porosity, which is not accessible to the consolidation agents. It can be concluded that, at the nanoscale, the microstructural characteristics of Maastricht limestone are preserved in the treated samples. The same slope of the high *Q* region of the SANS curve common to all samples is in agreement with a recent work^63^ in which, for the most deteriorated samples, the effect on fractal dimension on artificially weathered limestone was within the error of the measurement.

**Table 1:** SANS porosity (SP) from the fit of the SANS curves.

Sample	SP (%)
REF	0.200
E25	0.201
ARA_5	0.205
ARA_15	0.189
CAL_5	0.201
CAL_15	0.198
VAT_5	0.200
VAT_15	0.196

Data were averaged over two sample replicates. Not reported standard errors were below 1%.

Notably, differences with respect to the untreated sample are detected at the micrometric scale (accessed with SR-μCT) and not at the nanometric scale (accessed with SANS). This can be in part the consequence of the coarse microstructure of the Maastricht limestone and is also the indication that the consolidation treatment can produce new features at this scale. They include the new agglomerates of CaCO_3_ bridging between limestone grains observed with SEM (Figs 3 and S2–S4). This also confirms that the introduction of CaCO_3_ polymorphs is compatible with the treatment and improves its effectiveness in comparison with the application of nanolime alone. It must be pointed out that the evaluation of the performance of a consolidation treatment is a complex task. In fact, the effectiveness might be highly variable in function of the type of stone (in terms of mineralogy and pore network), the nature of the consolidation material, the solvent employed, the application method. Therefore, further studies are underway to test different treatment procedures, like brushing^11,76^ or application by capillarity^76^, modifying the solvent composition^4,6,19–21^ or applying water after the consolidation treatment^24^.

For the solution presented in this work, it can be can argued that improvements can be obtained also tailoring the grain size distribution of the synthetized CaCO_3_ to the pore network of the treated material. To this aim, optimization of the synthesis and milling steps will be considered.

## Conclusions

In present work, the application of nanolime suspensions in combination with three different synthetic CaCO_3_ polymorphs, aragonite, calcite and vaterite, were tested as consolidation treatment on weak highly porous Maastricht limestone. The changes of nano/microporosity before and after treatment were investigated using SR-μCT and SANS techniques. SEM observations revealed that the good chemical and structural compatibility of CaCO_3_ with nanolime and limestone, helped to effectively bound nanolime. In addition, the presence of CaCO_3_ particles improved the capability of nanolime to ‘bridge’ the grains of the limestone matrix. The penetration of CaCO_3_ particles was found to depend on their size and shape, being reduced in case of aragonite particles, which possess an elongated, needle-like shape. SR-μCT measurements evidenced a decrease in total porosity (open, as well closed porosity, not accessible to consolidation agents) in all treated specimens from 2 to 3% of the internal part of testing specimens. The lowest porosity values were detected for ARA_5, CAL_15, CAL_50 and VAT_15 consolidation mixtures. The effectiveness of the treatments with 15% of powder load were confirmed by a pore accessibility ratio investigation on the studied SR-μCT volumes. Only small changes in nanoporosity in the range from 80 to 200 nm were detected with SANS, which has been ascribed in part to the poor pore-filling capacity of nanoparticles in stones containing large porosity.

The presented results showed that in such substrates, the effectiveness of treatments with nanoparticles can be greatly enhanced by adding CaCO_3_ particles, although their morphological characteristics must be compatible with the stone pore network.

## Supplementary information

Supplementary Material

## Data Availability

All data will be available upon request.
