# Author Correction: Synthetic calcium carbonate improves the effectiveness of treatments with nanolime to contrast decay in highly porous limestone

**DOI:** 10.1038/s41598-021-03381-x

**Published:** 2021-12-14

**Authors:** Radek Ševčík, Alberto Viani, Dita Machová, Gabriele Lanzafame, Lucia Mancini, Marie-Sousai Appavou

**Affiliations:** 1 Institute of Theoretical and Applied Mechanics of the Czech Academy of Sciences, Prosecká 809/76, Praha 9, 190 00 Czech Republic; 2 Elettra-Sincrotrone Trieste S.C.p.A, SS 14- km 163.5, Area Science Park, 34149 Basovizza (Trieste), Italy; 3 Forschungszentrum Jülich GmbH, Jülich Centre for Neutron Science JCNS at MLZ, Lichtenbergstraße 1, 85747 Garching, Germany

Correction to: *Scientific Reports* 10.1038/s41598-019-51836-z, published online 24 October 2019

The Acknowledgements section in the original version of this Article was incomplete.

“This research was supported by the project from the Czech Science Foundation GA ČR grant 17-05030S. The authors would like to thank Roman Fabeš and Eva Pažourková for sample preparation. The authors acknowledge the CERIC-ERIC Consortium for the access to synchrotron radiation computed microtomography facilities at Elettra (Italy). This work is based on experiment performed at KWS-2 instrument operated by JCNS at the Heinz Maier-Leibnitz Zentrum (MLZ) in Garching (Germany).”

now reads:

“This research was supported by the project from the Czech Science Foundation GA ČR grant 17-05030S. The authors would like to thank Roman Fabeš and Eva Pažourková for sample preparation. The authors acknowledge the CERIC-ERIC Consortium for the access to synchrotron radiation computed microtomography facilities at Elettra (Italy). This work is based on experiment performed at KWS-2 instrument operated by JCNS at the Heinz Maier-Leibnitz Zentrum (MLZ) in Garching (Germany). The research leading to this result has been supported by the project CALIPSOplus under Grant Agreement 730872 from the EU Framework Programme for Research and Innovation HORIZON 2020.”

The original Article has been corrected.

