# Supplementary material for: Synthetic calcium carbonate improves the effectiveness of treatments with nanolime to contrast decay in highly porous limestone
Source: Sci Rep. 2019 Oct 24;9:15278. doi: 10.1038/s41598-019-51836-z (PMC6813344; doi:10.1038/s41598-019-51836-z)
Supplement: Supplementary file 1 — Supplementary Material [file 41598_2019_51836_MOESM1_ESM.pdf]

Synthetic calcium carbonate improves the effectiveness of treatments with nanolime to contrast decay in highly porous limestone

Radek Ševčík[a*], Alberto Viani[a], Dita Machová[a], Gabriele Lanzafame[b], Lucia Mancini[b], Marie-Sousai Appavou[c]

[a]Institute of Theoretical and Applied Mechanics of the Czech Academy of Sciences, Prosecká 809/76, Praha 9, 190 00, Czech Republic.

[b]Elettra-Sincrotrone Trieste S.C.p.A., SS 14- km 163.5, Area Science Park, 34149 Basovizza (Trieste), Italy.

[c]Forschungszentrum Jülich GmbH, Jülich Centre for Neutron Science JCNS at MLZ, Lichtenbergstraße 1, 85747 Garching, Germany

*Corresponding author

Radek Ševčík, PhD. E-mail: sevcik@itam.cas.cz; Tel. +420 567225322;

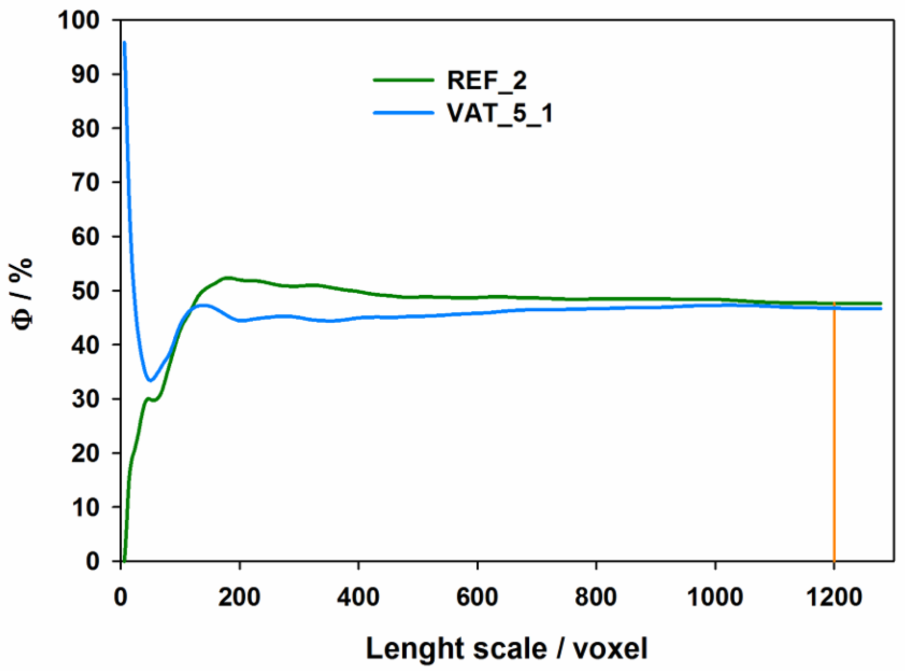

Fig. S1: Example of relationship between porosity and scale of measurement for two samples, as indicated. Vertical orange bar marks the choice of the REV, corresponding to a volume of 1200x1200x1200 voxels. A larger VOI (1800x1800x1948 voxels) has been adopted in the analysis of SR-μCT data.

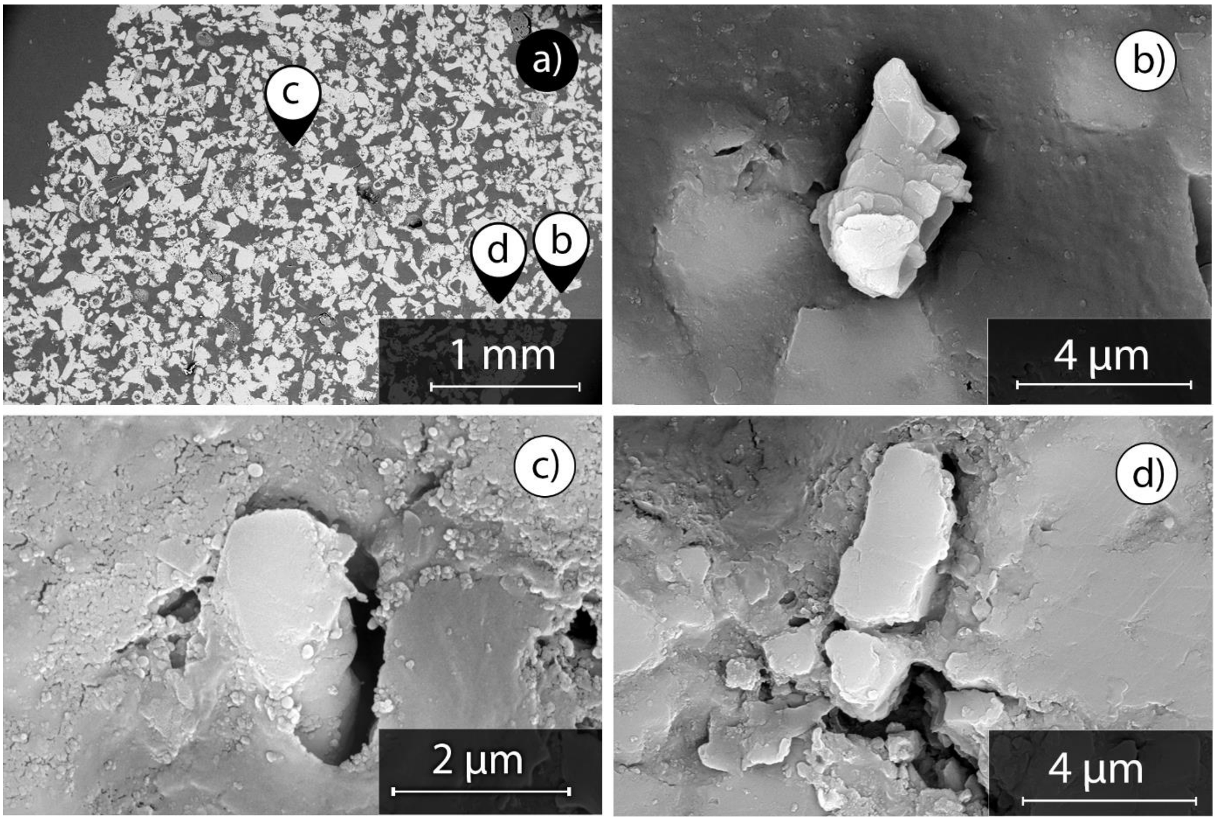

Fig. S2: SEM images in cross-section of ARA50 sample. The highlights points with assigned letters in Fig. 1Sa correspond to the positions of detected synthetic aragonite particles within consolidated Maastricht limestone.

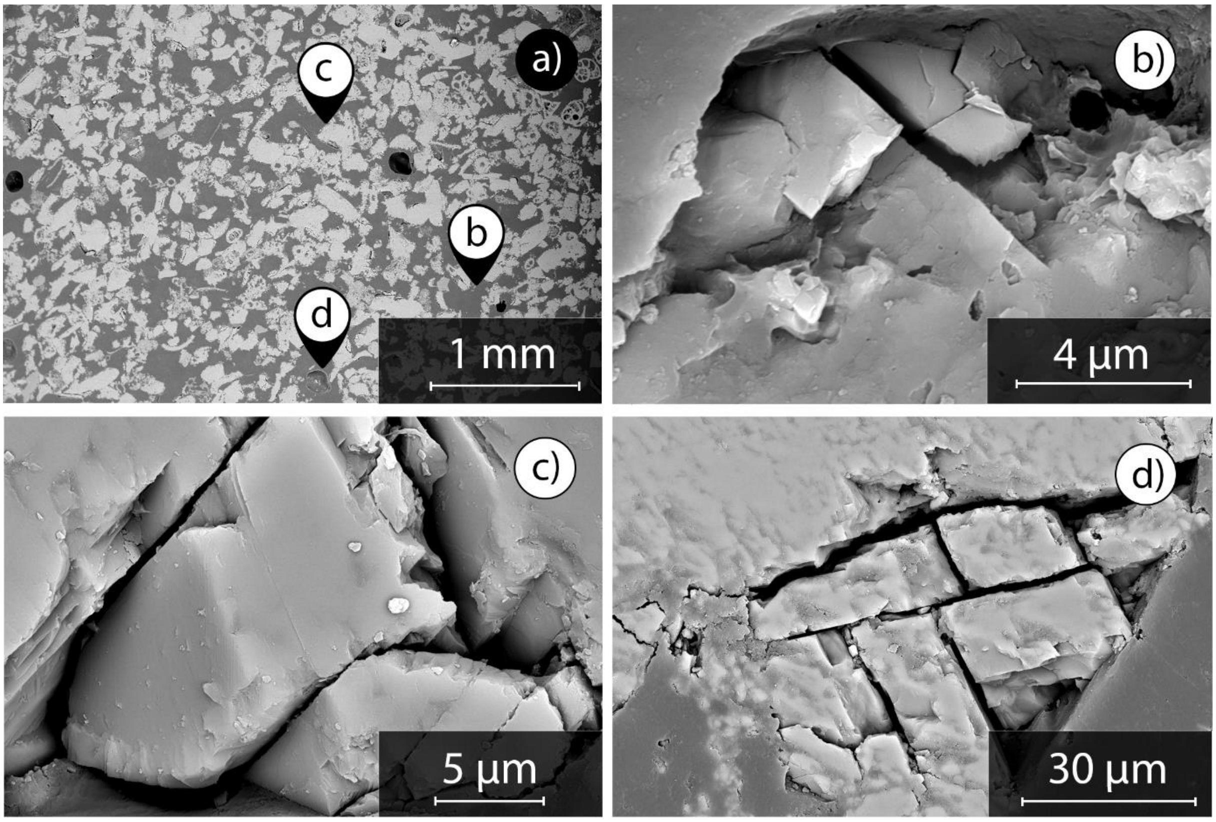

Fig. S3: SEM images in cross-section of CAL50 sample. The highlights points with assigned letters in Fig. 2Sa correspond to the positions of detected synthetic calcite particles within consolidated Maastricht limestone.

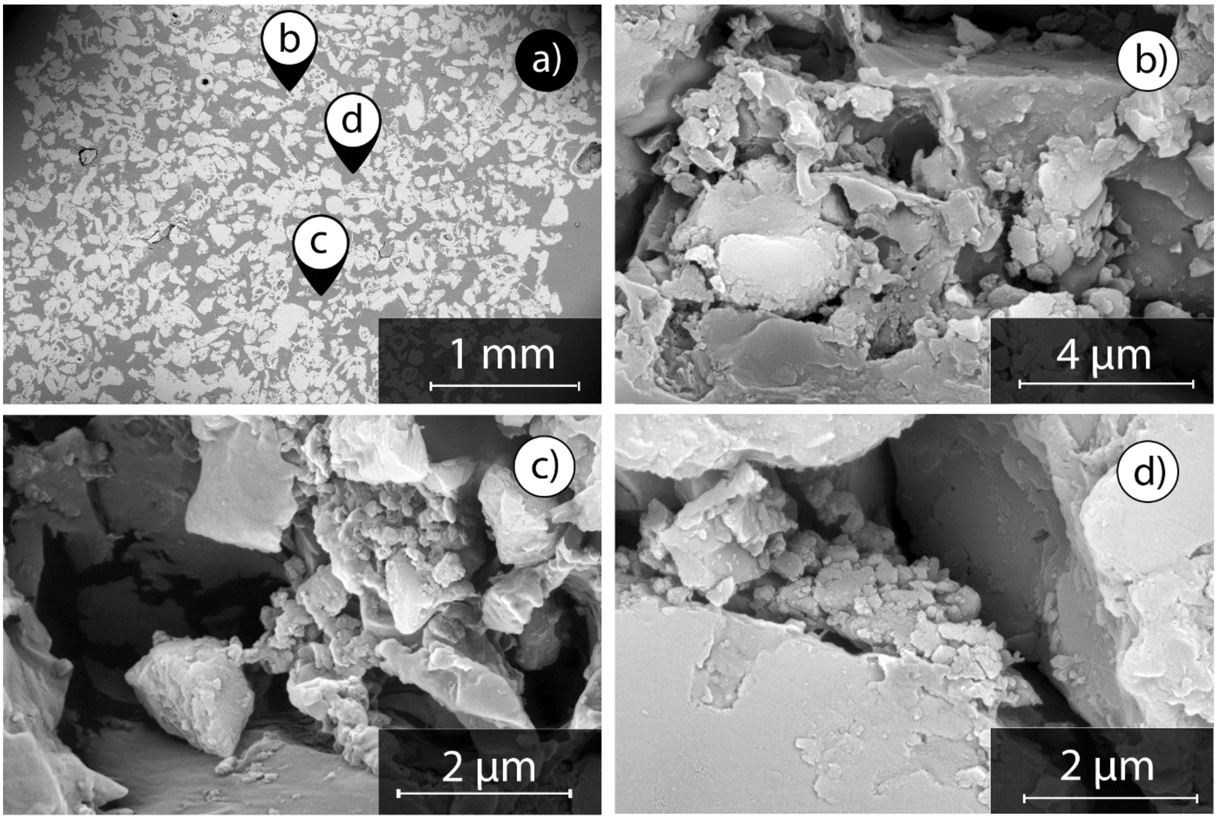

Fig. S4: SEM images in cross-section of VAT50 sample. The highlights points with assigned letters in Fig. 3Sa correspond to the positions of detected synthetic vaterite particles within consolidated Maastricht limestone.

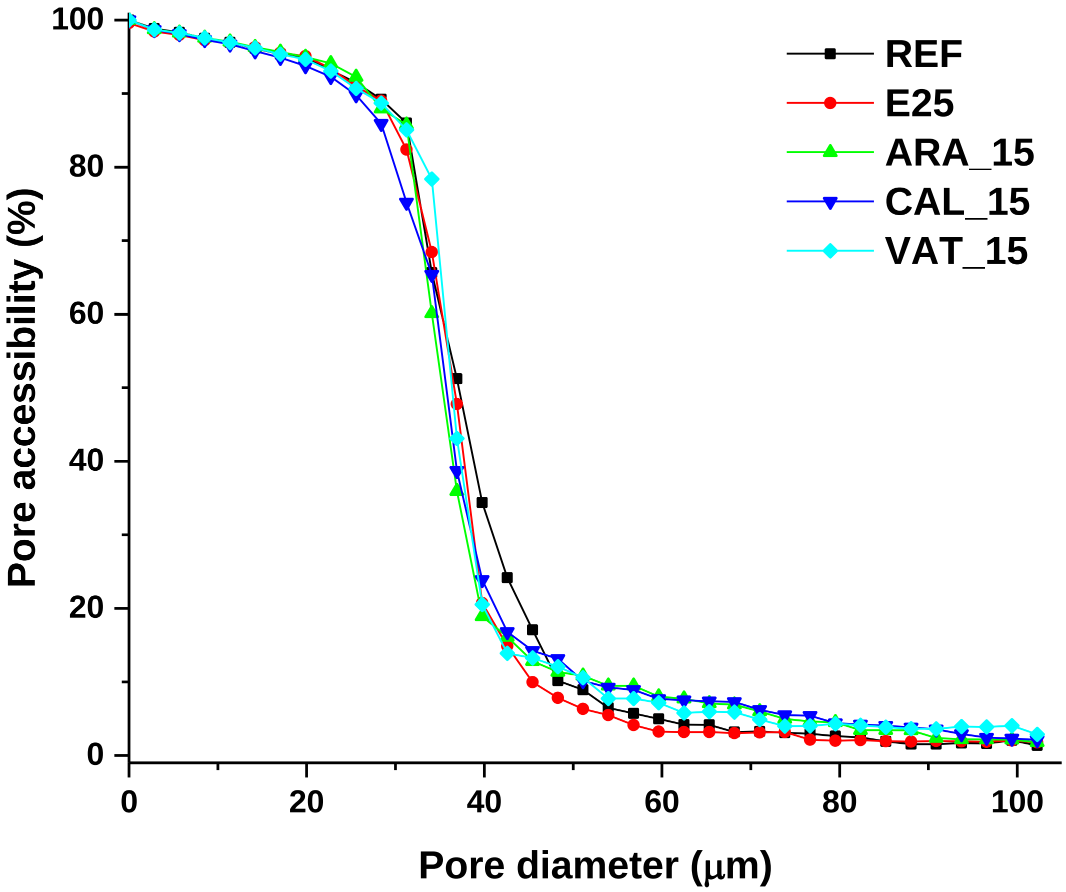

Fig. S5: Pore accessibility ratio of the intra-aggregate pore space for the spherical entities.

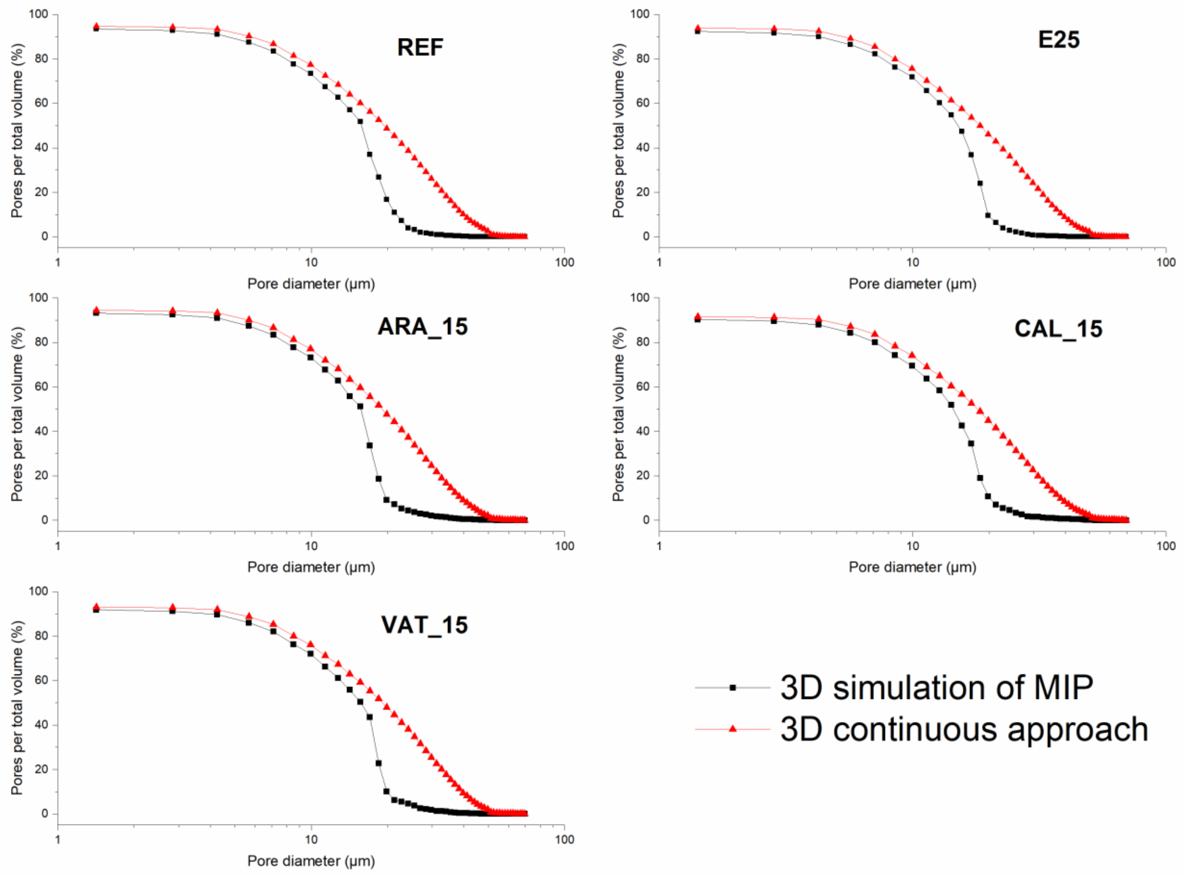

Fig. S6: Comparison of the cumulative distribution function of intra-aggregate porosity calculated for investigated samples based on the 3D simulation of MIP and 3D continuous approach

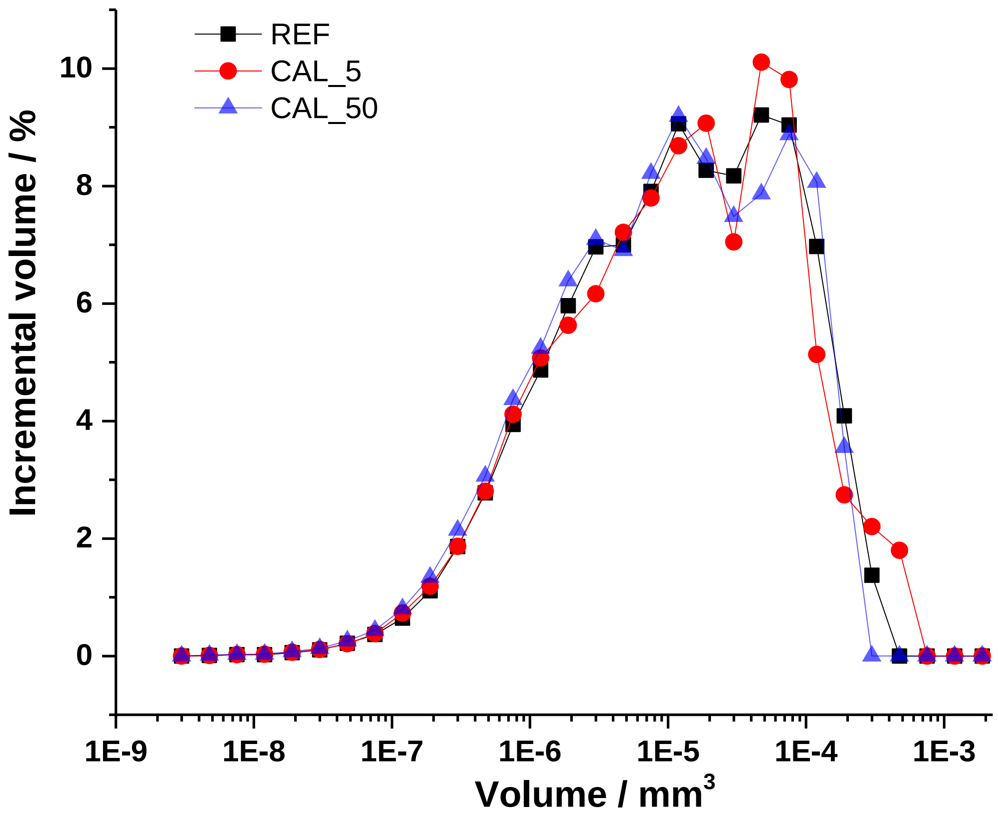

Fig. S7: Pore-size distribution frequency from SR-µCT data for some of the measured samples, as indicated. The range on the large pore volume side has been limited for sake of clarity.

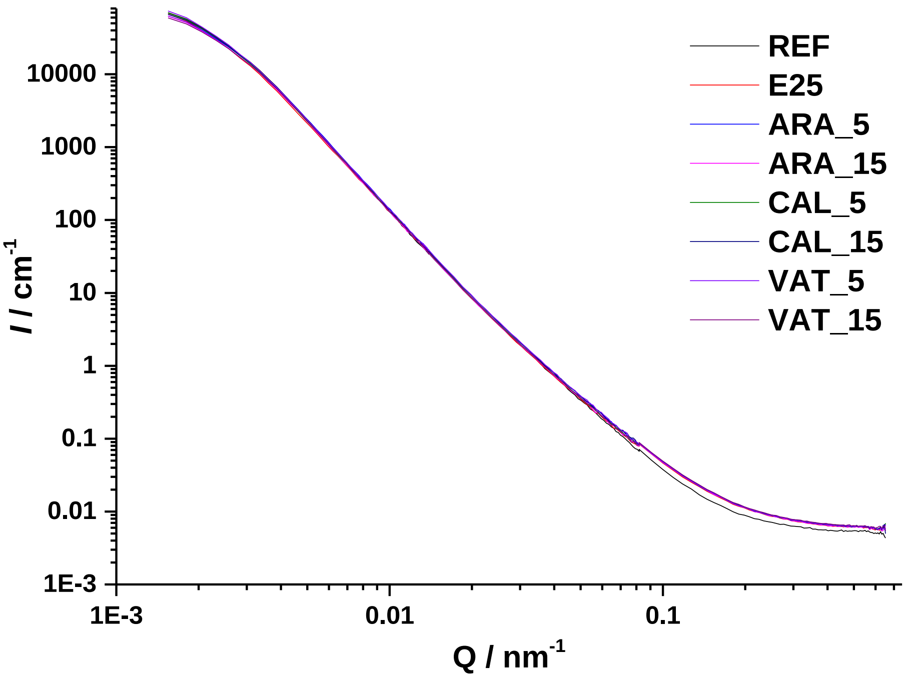

Fig. S8: Normalized SANS curves of the untreated sample of Maastricht limestone and samples treated with different mixtures of consolidate agents, as indicated.

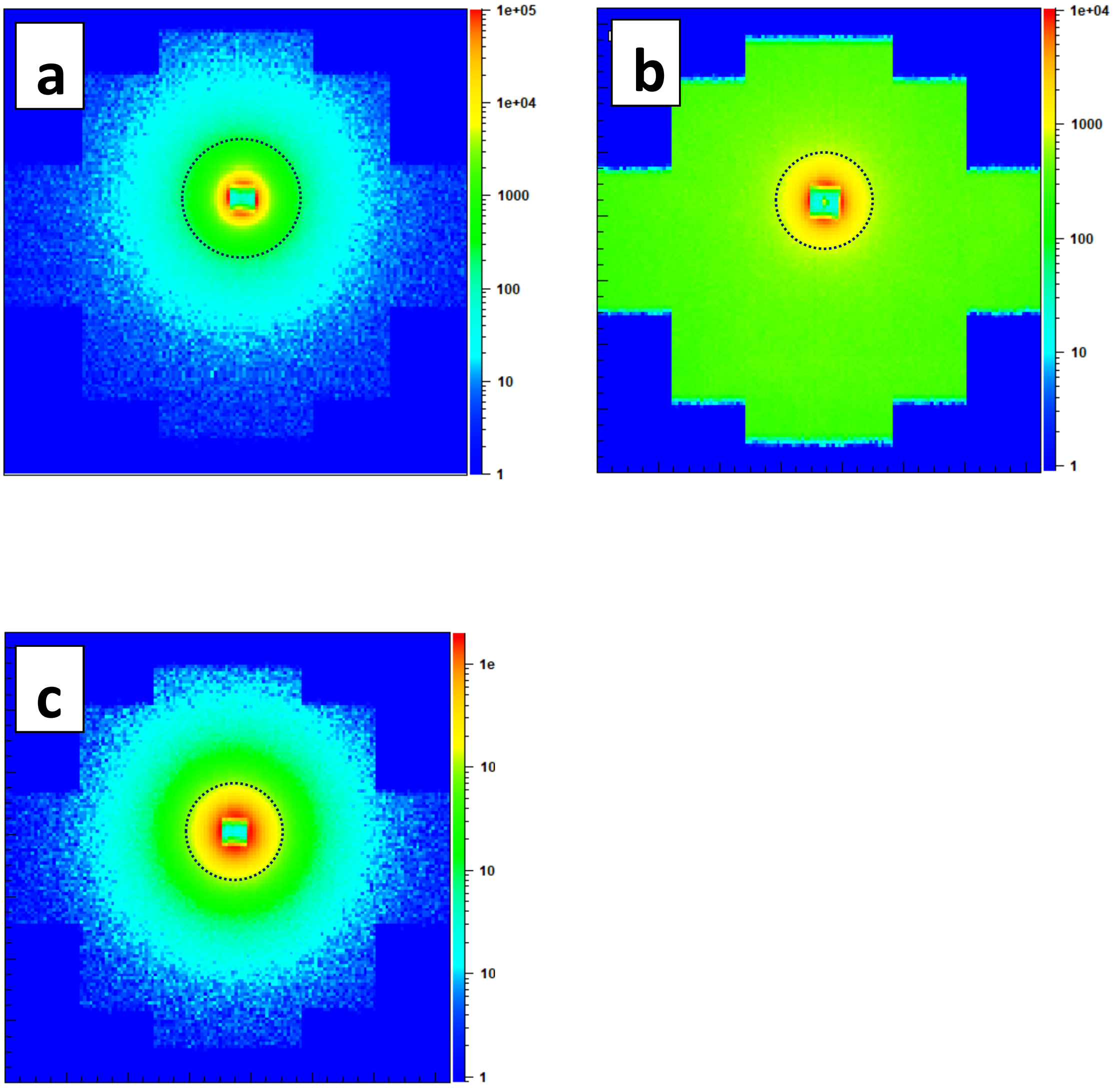

Fig. S9: 2-D SANS spectra in the low- (**a**), intermediate (**b**) and high-*Q* (**c**) range, corresponding to the three detector positions, for one of the reference samples. The distance from the center is proportional to the scattering vector *Q*. Color scale is adopted to illustrate the scattered intensity. Circular dotted lines are drawn as guides for the eyes in order to appreciate the isotropy of the 2-D signal.

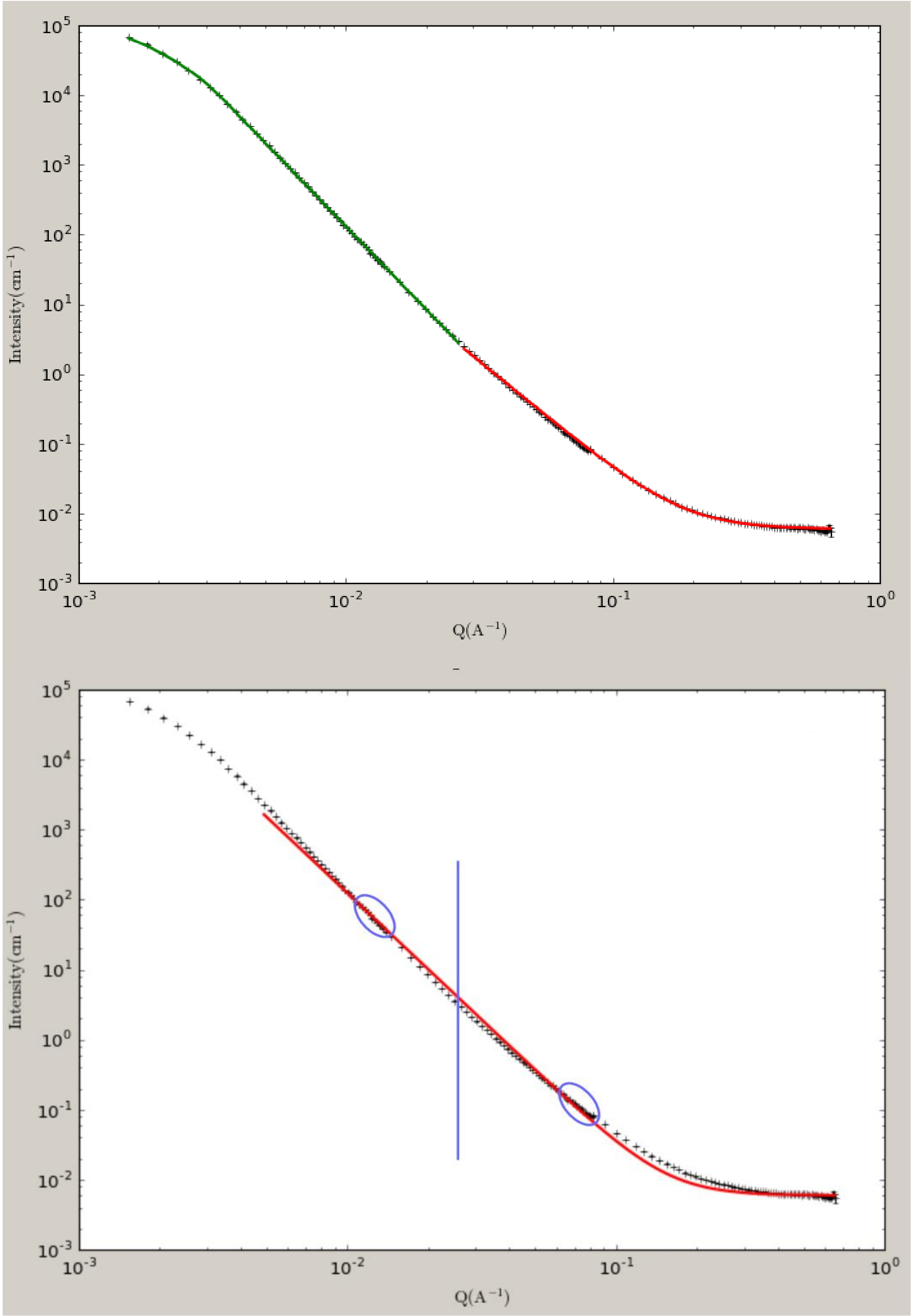

Fig. S10: Example of fit of the SANS curve for sample E25_1 adopting a two-slope model, including the Guinier regime at low-*Q* (top) and a single-slope model (bottom). Data points include vertical bars corresponding to the estimated standard deviation. Blue ellipses indicate the regions where data points from 2 different detector positions are merged together. Blue vertical bar indicates the value of *Q* refined during the fit of the two-slope model, locating the slope change.
